# Tumefactive Multiple Sclerosis Mimicking a High-Grade Glioma: A Case Report and Literature Review

**DOI:** 10.3390/reports9020188

**Published:** 2026-06-16

**Authors:** Maria P. Fernandez-Gomez, Luis Rafael Moscote-Salazar, Jesus Francisco Saltaren Fonseca, Guillermo de Jesus Aguirre Vera, Willem Calderon Miranda, Jose Valerio

**Affiliations:** 1 Neurosurgery Oncology Department, Latinoamerica Valerio Foundation, Weston, FL 33332, USA; mariapaulafg96@gmail.com (M.P.F.-G.); rafaelmoscote21@gmail.com (L.R.M.-S.); jesus.saltaren@gmail.com (J.F.S.F.); jevalerio@jvaleriomd.com (J.V.); 2 Section of Neuroradiology, Hospital Universitari de Vall d’Hebron, 08035 Barcelona, Spain; willem.calderon@hotmail.com; 3 Neurosurgery Oncology, Neurosurgery Oncology Center of Excellence, Miami Neuroscience Center, Larkin Healthcare System, Miami, FL 33143, USA; 4 Neurological Surgery, Palmetto General Hospital, Healthcare Systems of America, Hialeah, FL 33016, USA

**Keywords:** tumefactive multiple sclerosis, multiple sclerosis, tumefactive demyelinating lesions, demyelinating disease, horseshoe sign

## Abstract

**Background and Clinical Significance:** Tumefactive Multiple Sclerosis (TMS) represents a rare and diagnostically challenging form of demyelinating disease characterized by large space-occupying lesions that can closely mimic intracranial neoplasms, abscesses, and other inflammatory or vascular conditions. **Case Presentation:** The case highlights the overlapping radiologic features that frequently lead to diagnostic uncertainty and underscores the importance of careful interpretation of multimodal imaging and ancillary studies. Overall a comprehensive multidisciplinary evaluation is essential to reduce the risk of misdiagnosis and avoid unnecessary invasive interventions. **Conclusions:** This review summarizes current evidence regarding the diagnostic approach, imaging characteristics, and therapeutic strategies for tumefactive demyelinating lesions. Additionally, we present a clinical case that illustrates the diagnostic complexity of this entity, in which neuroimaging findings and cerebrospinal fluid analysis supported a demyelinating rather than neoplastic process.

## 1. Introduction and Clinical Significance

Tumefactive Multiple Sclerosis (TMS) is a rare variant of multiple sclerosis (MS) characterized by solitary or multiple brain lesions measuring 2 cm or greater in diameter [1,2,3,4]. These lesions have a pseudotumoral appearance [5] and are typically larger than the demyelinating plaques commonly observed in classical MS. As a result, they often mimic intracranial neoplasms, infections, or other non-demyelinating brain pathologies [6,7]. TMS accounts for approximately 2.8–7% of all MS cases [1,6].

On magnetic resonance imaging (MRI), these lesions frequently demonstrate ring-enhancing patterns [4] and surrounding perifocal edema [7,8,9]. Additional imaging findings may include reduced cerebral blood volume [3], hypervascularity, increased vascular permeability, and varying degrees of mass effect. These characteristics can significantly complicate the differential diagnosis, particularly when distinguishing TMS from cerebral abscesses or primary brain neoplasms [2,7].

The lesions observed in this condition are commonly referred to as “Tumefactive Demyelinating Lesions” (TDLs) [9], a term best understood within the broader spectrum of MS pathology [10]. They are most frequently located in the cerebral white matter, especially within the frontal and parietal lobes, although they may occur in any region of the central nervous system (CNS). Less common tumefactive variants of MS include Schilder’s disease, acute Marburg MS, and Balo’s concentric sclerosis [8].

When TDLs present as solitary lesions, the diagnostic process becomes particularly challenging due to their radiologic resemblance to intracranial neoplasms. This similarity may lead to misdiagnosis and, consequently, unnecessary invasive procedures or treatments that carry potential risks for the patient [8]. To reduce diagnostic uncertainty, several advanced imaging modalities have been investigated, including diffusion-weighted imaging (DWI), magnetic resonance spectroscopy (MRS), fluorodeoxyglucose positron emission tomography (FDG-PET) [2,6,8], cerebrospinal fluid (CSF) analysis [7], and, when necessary, brain biopsy, which remains the gold standard for definitive diagnosis [7].

The clinical significance of TMS lies in its frequent radiologic overlap with high-grade gliomas, lymphomas, abscesses, and other space-occupying lesions, which may lead to misdiagnosis, delayed immunotherapy, or unnecessary invasive procedures. Prompt recognition through careful integration of imaging findings, cerebrospinal fluid findings, and clinical evolution is therefore essential.

## 2. Case Presentation

A 25-year-old right-handed woman with a history of heroin and cocaine use and no other known medical conditions presented with the acute onset of reading difficulty, anomia, right hemisensory loss, and right hemiparesis. She denied headache, nausea, or vomiting.

On neurological examination, she was alert and oriented to person, place, and time. Language assessment revealed intact comprehension and naming, although her speech was mildly slowed and deliberate. Sensory testing demonstrated decreased light-touch sensation over the right hemibody, along with right-sided astereognosis. Cranial nerve examination showed mild resting asymmetry of the right face, with preserved voluntary facial movements.

Motor examination revealed increased tone and reduced strength (Medical Research Council grade 4−/5) in the right upper and lower extremities, while strength on the left side was normal. Deep tendon reflexes were brisk (3/4) and symmetric, with flexor plantar responses. Rapid alternating movements were markedly slowed on the right. She required significant assistance for ambulation. Babinski responses were absent bilaterally.

Magnetic resonance imaging (MRI) of the brain demonstrated a large T2/FLAIR hyperintense lesion within the left cerebral hemisphere, associated with surrounding vasogenic edema and mass effect (Figure 1). Given its size and imaging characteristics, a high-grade glioma was initially considered the leading diagnosis. However, post-contrast sequences revealed an incomplete “open-ring” enhancement pattern, also referred to as the “horseshoe sign”, with the open portion directed toward the cortical surface. This finding strongly favored tumefactive demyelination over a neoplastic process.

Cerebrospinal fluid analysis revealed clear CSF containing 0 erythrocytes and 1 leukocyte/µL (78% lymphocytes). Glucose was 75 mg/dL, and protein was 34 mg/dL. Immunologic studies demonstrated a mildly elevated IgG index (0.72; reference range, 0.0–0.62) and increased CNS IgG synthesis (4.9 mg/day; reference range, 0–3.0). An isolated atypical CSF-restricted oligoclonal band was identified, with no apparent corresponding abnormality in the serum. HIV testing was negative (Table 1).

In light of the 2024 revisions to the McDonald diagnostic criteria, additional MRI biomarkers such as the central vein sign (CVS) and paramagnetic rim lesions (PRLs) may provide supportive evidence and increase diagnostic specificity for multiple sclerosis when available. In this case, however, the available MRI protocol did not include susceptibility-sensitive sequences optimized for reliable assessment of CVS or PRLs, such as susceptibility-weighted imaging, phase imaging, or quantitative susceptibility mapping. Therefore, these imaging biomarkers could not be retrospectively evaluated and were not used to support the diagnosis. Instead, the diagnosis was supported by the open-ring enhancement pattern, absence of prominent central diffusion restriction, cerebrospinal fluid inflammatory findings, and multidisciplinary clinicoradiologic interpretation [10].

The convergence of these radiographic and cerebrospinal fluid abnormalities is most consistent with a tumefactive demyelinating process rather than a primary glial neoplasm.

## 3. Discussion

This case highlights the diagnostic complexity of tumefactive multiple sclerosis, particularly when neuroimaging findings initially suggest a high-grade neoplasm. The presence of open-ring enhancement, supportive cerebrospinal fluid findings, and multidisciplinary interpretation were critical in favoring a demyelinating process. The following discussion places this case in the context of the current literature, emphasizing the main diagnostic pitfalls, imaging clues, therapeutic considerations, and prognostic implications of tumefactive demyelinating lesions.

### 3.1. Epidemiology

The prevalence of TMS is estimated to range between 1 and 2 cases per 1000 patients diagnosed with MS [9,10,11,12]. However, the exact incidence remains uncertain. According to the available literature, it has been described as approximately 3 cases per 1,000,000 inhabitants, with a higher predominance in females.

Late-onset MS is relatively uncommon, representing approximately 0.6–0.75% of cases diagnosed after the sixth decade of life. This demographic factor may further complicate the distinction between TDLs and primary brain tumors in older patients [11].

TDLs appear to be reported more frequently in Asian populations, with incidence rates ranging from 6.3% to 11.76%, a finding that may reflect ethnic and immunogenetic heterogeneity [10]. In 2013, Kuan et al. analyzed 190 patients diagnosed with MS in Taiwan between 1985 and 2010, of whom 12 were confirmed to have TMS, corresponding to approximately 6.3% of the studied cohort. Importantly, this figure does not represent national prevalence, as the study population was derived from a single medical center in Taipei [13].

### 3.2. Etiology

The underlying causes of tumefactive demyelinating lesions (TDLs) remain incompletely understood. However, a potential association has been proposed between immature isoforms of myelin basic protein (MBP) and the development of extensive, atypical demyelinating lesions, as described in acute Marburg multiple sclerosis. This observation suggests that alterations in MBP structure or processing may contribute to the pathogenesis of TDLs.

Different MBP isoforms play distinct roles in normal myelin maintenance, and increased susceptibility to protein degradation may promote inflammatory activity and potentially increase the risk of TDL recurrence [10].

### 3.3. Localization

TDLs most commonly arise within the cerebral hemispheres, particularly in the frontal and parietal lobes [5]. Nevertheless, other regions of the central nervous system (CNS) may also be affected, including segments of the cervical spinal cord [3].

Kuan et al. reported that approximately 80% of lesions were located supratentorially, predominantly in periventricular and juxtacortical regions, while 40% showed spinal cord involvement [13].

### 3.4. Clinical Manifestations

The clinical presentation of patients with TMS can be polysymptomatic, particularly when lesions are large and multiple [8]. Symptoms arising from TDLs may present acutely or subacutely [1,2,3,4,5], depending on the location, size, and associated mass effect of the lesion [5,8]. Clinical manifestations of TMS include headache, cognitive abnormalities, mental confusion, seizures, apraxia, aphasia [1,3,4], increased intracranial pressure, vomiting, behavioral changes, visual disturbances, optic neuritis, and hemiparesis [3]. TDLs frequently cause memory dysfunction, Gerstmann syndrome, and, in some patients, encephalopathies [9]. Visual and somatosensory evoked potentials are abnormal in 33–60% of cases diagnosed with TMS [1].

In general, these signs and symptoms are not typically associated with MS, which often leads to TMS being overlooked in the differential diagnosis and mistaken for neoplasms or infections. However, even in patients with a preexisting diagnosis of MS, the possibility of a neoplasm or other non-demyelinating pathology cannot be excluded, which often justifies performing a biopsy to establish a definitive diagnosis [11].

### 3.5. Diagnosis

Histopathologically, TDLs may resemble neoplasms due to features such as hypercellularity, gliosis, atypical reactive astrocytes, mitotic activity, and perivascular lymphocytic infiltration [6,11], as well as areas of necrosis and cystic change [11], and the presence of Creutzfeldt cells [9]. In addition, numerous foamy macrophages containing myelin debris are typically observed, along with relative preservation of axons [11].

Although biopsy provides a definitive diagnosis, tissue sampling carries inherent risks. It has been reported that biopsy-related trauma may result in neurological deficits and, in rare cases, even death [11]. Nevertheless, in patients with diagnostic uncertainty and complex clinical presentations, biopsy is often justified to clarify the underlying pathology. The rate of misdiagnosis of TDLs has been estimated at approximately 31% [9], potentially leading to unnecessary interventions or inappropriate treatments. Distinguishing TDLs from neoplasms is therefore essential, particularly to avoid unwarranted brain irradiation, which may exacerbate demyelinating disease [8,11].

In a case report by Hamed, the diagnosis of TMS was based on several clinical and radiologic criteria, including acute symptom onset, cognitive deterioration, multiple bilateral lesions on imaging, absence of cortical involvement, minimal or absent mass effect, gadolinium enhancement, regression following corticosteroid therapy, and no evidence of new lesions on follow-up MRI [1].

### 3.6. Differential Diagnosis

The diagnosis of TDLs in the context of MS remains challenging because of their radiologic resemblance to neoplasms, abscesses, acquired demyelinating disorders, and vascular lesions [3]. To improve diagnostic accuracy and reduce unnecessary biopsies that may expose patients to avoidable risks, several advanced imaging techniques have been explored.

Magnetic resonance spectroscopy (MRS) is a non-invasive modality that evaluates biochemical changes in intracranial lesions and has been identified in the literature as a valuable tool for distinguishing Tumefactive Multiple Sclerosis (TMS) from primary brain tumors. MRS analyzes metabolites such as N-acetyl aspartate (NAA), choline, creatine, lipids (Lp), and lactate (Lac). Decreased NAA levels are associated with neuronal and axonal loss as well as mitochondrial dysfunction, whereas elevated choline, Lp, and Lac levels may reflect active demyelination or increased cellular turnover. Kobayashi et al. reported that in two of three TMS cases, NAA levels were reduced and, in all three cases, the choline/creatine ratio was elevated in peripheral regions consistent with myelin degradation [2].

It is important to recognize that patients with MS may also develop gliomas, which can further complicate interpretation of imaging findings. In some cases, TMS and gliomas may coexist, adding another layer of diagnostic complexity [9]. Butteriss et al. described cases initially interpreted as TMS on preoperative MRI that were later confirmed as oligodendrogliomas following surgical resection [8]. A favorable response to corticosteroids [6], along with progressive reduction in lesion size on serial neuroimaging, strongly supports a demyelinating tumefactive lesion rather than a neoplastic or abscess etiology [3]. Nevertheless, relying exclusively on MRS or PET findings may be insufficient. Careful longitudinal MRI follow-up, with or without corticosteroid therapy, is often necessary to establish diagnostic confidence [8].

Although cerebrospinal fluid (CSF) analysis has not demonstrated strong independent diagnostic value for TMS, the presence of oligoclonal bands and elevated immunoglobulin G levels supports an underlying inflammatory demyelinating process [9].

### 3.7. When to Consider Brain Biopsy

Brain biopsy remains a procedure of last resort in the evaluation of TDLs. It should be considered when the patient’s clinical history, imaging findings, and laboratory studies are inconclusive or when there is a strong suspicion of an alternative diagnosis such as neoplasm or infection. Given the inherent risks associated with intracranial tissue sampling, biopsy must be undertaken cautiously and reserved for cases with clear indications or after failure of first-line medical therapies [14].

### 3.8. Atypical Imaging Features Suggestive of Alternative Pathology

When MRI findings are atypical or do not support a demyelinating pattern, biopsy should be more strongly considered. Concerning features include homogeneously enhancing mass-like lesions, thick-walled or complete ring enhancement with irregular margins, disproportionate mass effect or vasogenic edema, and marked central diffusion restriction—findings more commonly associated with high-grade glioma, primary CNS lymphoma, or cerebral abscess [14].

Furthermore, dynamic susceptibility contrast MRI may reveal elevated perfusion within lesions exhibiting hemorrhagic components or necrotic cores. Such features suggest a more aggressive process and may warrant early tissue diagnosis to exclude neoplastic or infectious etiologies [15].

### 3.9. Lack of Clinical or Radiologic Improvement Following Steroid Therapy

A defining characteristic of demyelinating lesions is their responsiveness to high-dose corticosteroids [8]. Lesions that fail to regress or that enlarge despite appropriate steroid therapy, raise concern for alternative, non-inflammatory etiologies. Ongoing clinical deterioration, including worsening neurological deficits, increased intracranial pressure, or rapid lesion expansion despite treatment, should prompt consideration of expedited biopsy to establish a definitive diagnosis and guide management [16].

### 3.10. Absence of Supportive Ancillary Markers for Demyelination

The likelihood of an inflammatory demyelinating process decreases when cerebrospinal fluid (CSF) analysis is negative for oligoclonal bands and shows a normal IgG index, and when serum testing is negative for AQP4-IgG and MOG-IgG. In addition, patients without typical radiologic features of MS—such as periventricular or callosal lesions—and older individuals without a prior history of demyelinating events may lack sufficient ancillary evidence to support a diagnosis of MS. In such cases, particularly when imaging findings remain inconclusive or atypical, brain biopsy may still be warranted [17].

### 3.11. Metabolic Imaging and Advanced Diagnostics to Guide Biopsy Decisions

When diagnostic uncertainty persists, additional imaging modalities may provide further clarification. Among these, ^18F-FDG PET can offer useful metabolic information. Tumefactive demyelinating lesions typically demonstrate hypometabolism, whereas high-grade neoplasms are more often characterized by increased metabolic activity [16].

If reduced metabolic activity on PET is accompanied by hypoperfusion on MRI, the findings favor a demyelinating etiology, and biopsy may reasonably be deferred in clinically stable patients. Conversely, elevated metabolic activity, even in the absence of overt hyper perfusion, should heighten suspicion for a neoplastic process and may justify early neurosurgical consultation and consideration of tissue diagnosis [18].

### 3.12. Tumefactive Variants of Multiple Sclerosis

Less common tumefactive variants of multiple sclerosis (MS) include acute Marburg MS, Schilder’s disease, and Balo’s concentric sclerosis.

Acute Marburg MS was first described in 1906 by Otto Marburg as a severe, idiopathic inflammatory demyelinating disease of the central nervous system (CNS) characterized by large, rapidly progressive lesions. Histopathologically, it is marked by prominent macrophage infiltration, extensive demyelination, axonal loss, and areas of necrosis. Alterations in the myelin basic protein (MBP) gene have been reported, potentially contributing to structural instability of myelin [9]. Clinically, this variant is associated with aggressive axonal injury, rapid neurological deterioration, and, in some cases, fatal outcomes [1].

Schilder’s disease, also referred to as myelinoclastic diffuse sclerosis, was first described in 1912 as a demyelinating disorder characterized by large bilateral cerebral lesions. Its pathogenesis remains incompletely understood, and historically, the term has occasionally been applied to X-linked adrenoleukodystrophy, contributing to some diagnostic ambiguity.

Balo’s concentric sclerosis is distinguished by concentric lamellar patterns of demyelination [1]. On MRI, it typically demonstrates ring-shaped areas of T2 hyperintensity alternating with bands of relatively preserved or partially remyelinated tissue, corresponding to regions of T2 isointensity. Lesions are most commonly located in the basal ganglia and cerebellum, although involvement of the spinal cord and optic nerve has also been described [9].

### 3.13. Imaging Findings

Several neuroimaging modalities have been proposed to aid in the diagnosis of TMS, including magnetic resonance spectroscopy (MRS), diffusion-weighted imaging (DWI), perfusion-weighted imaging (PWI), and apparent diffusion coefficient (ADC) analysis [6]. TMS presents significant diagnostic challenges on imaging, as associated mass effect and perilesional edema can closely resemble brain tumors or infections. Nonetheless, the presence of ring enhancement following gadolinium administration is considered suggestive of a tumefactive demyelinating lesion (TDL) [7].

Comparative imaging studies have shown that when MRI-enhancing regions are correlated with corresponding areas on CT, hypoattenuation may help differentiate TDLs from gliomas or lymphomas, although this distinction appears to be most reliable for these two tumor subtypes. In contrast to cerebral abscesses, TMS lesions generally appear centrally homogeneous on T2-weighted images [6]. Furthermore, when compared with abscesses and primary brain tumors, the degree of mass effect and surrounding edema in inflammatory demyelinating disease (IDD) is often proportionally smaller relative to lesion size. Indeed, the relative lack of mass effect has been proposed as a distinguishing feature of MS plaques when compared to other space-occupying lesions [11].

Although no imaging finding is pathognomonic for TDLs, certain features may support the diagnosis. These include a T2-hypointense rim surrounding the lesion and the presence of additional white matter plaques typical of MS [9]. While contrast enhancement is frequently observed in TMS lesions, some cases demonstrate no enhancement, indicating that the absence of contrast uptake does not exclude a TDL [12]. Other MRI characteristics may include vascular structures traversing the center of the lesion on T2-weighted images and markedly reduced cerebral blood flow compared with normal white matter [6].

DWI assesses the diffusion of water molecules within tissue. Due to the heterogeneous pathological composition of MS lesions, ADC values may vary considerably [2]. In contrast to primary CNS lymphomas, which typically demonstrate restricted diffusion (hyperintense on DWI and hypointense on ADC maps), demyelinating lesions more often exhibit increased DWI signal intensity with elevated ADC values. However, peripheral diffusion restriction with lower ADC values can occasionally be observed in acute demyelinating lesions [6].

MRS provides additional metabolic information that may improve diagnostic specificity. N-acetylaspartate (NAA) serves as a marker of neuronal integrity in MS, whereas elevated choline peaks reflect increased membrane turnover associated with active demyelination and inflammation [4]. TDLs are commonly associated with elevated choline, glutamate–glutamine complexes, and lactate levels. The combination of increased lactate, reduced NAA/creatine ratios, and elevated choline/creatine ratios generally supports an inflammatory or demyelinating process [5,7]. However, TDLs may occasionally demonstrate metabolic patterns similar to those seen in brain tumors, including increased choline/creatine and decreased NAA/creatine ratios, which can limit the diagnostic utility of MRS alone. In such cases, fluorodeoxyglucose positron emission tomography (FDG-PET) may provide complementary diagnostic information [9].

### 3.14. Proposed TDLs Classification

Title: Valerio Classification of Tumefactive Multiple Sclerosis (MS)Purpose: Stratify tumefactive demyelinating lesions (TDLs) based on MRI characteristics, clinical course, and likelihood of conversion to classical MS.
**Type**

**MRI Features**

**Clinical Features**

**Prognostic Implications**
Type I—Classic Tumefactive LesionSingle lesion ≥ 2 cm, open-ring enhancement, minimal mass effectSubacute onset (weeks), mild to moderate focal deficitsTypically responds to corticosteroids; low risk of tumor misdiagnosisType II—Aggressive/Atypical LesionLarge lesion > 4 cm, closed/nodular ring enhancement, significant mass effect or edemaRapid onset (<7 days), severe neurological deficitsHigher risk of misdiagnosis with glioma or lymphoma; consider early biopsy if no response to steroidsType III—Multiple Lesions/Recurrent TDLsMultiple lesions in different brain regions, variable enhancementRecurrent episodes or multifocal deficitsHigher likelihood of progression to classical MS or MOGAD; close follow-up requiredType IV—Radiologically Isolated TDLLesion discovered incidentally or on surveillance MRI, minimal edema, subtle enhancementClinically silentMonitor closely; conversion risk uncertain, may not require immediate therapyNotes: Open-ring enhancement favors demyelination, whereas closed/nodular ring favors neoplasm.Mass effect and edema correlate with acute severity and urgency of intervention.Clinical correlation is essential; early corticosteroid response can guide diagnosis.This classification is useful for guiding follow-up, treatment intensity, and decisions regarding biopsy.

### 3.15. Treatment

Currently, there is no standardized immunomodulatory regimen specifically established for TMS. Reported pharmacologic options include methylprednisolone, beta interferons, plasma exchange, rituximab, and natalizumab [1,9]. However, high-dose corticosteroid therapy remains the first-line treatment [9], as most patients demonstrate a favorable clinical and radiologic response [6], occasionally obviating the need for surgical intervention [19].

In patients who fail to respond adequately to corticosteroids, plasma exchange (PLEX) is commonly employed. If clinical improvement remains insufficient, escalation to therapies such as rituximab or cyclophosphamide may be considered. Conversely, some studies have advised caution with fingolimod due to reports of tumefactive demyelinating lesions (TDLs) associated with its use [8].

#### 3.15.1. Acute Phase: Containment and Stabilization

High-dose intravenous corticosteroids constitute the cornerstone of acute management. A typical regimen consists of 1 g of methylprednisolone daily for 3 to 5 days. Corticosteroid therapy often leads to marked radiologic improvement, sometimes within weeks, including reduction in lesion volume, contrast enhancement, and surrounding vasogenic edema [20,21]. This response is particularly evident in steroid-responsive inflammatory demyelination.

In cases of partial or absent response, plasma exchange should be considered. A standard PLEX course typically includes 5 to 7 exchanges over 10 to 14 days. Evidence suggests benefit in steroid-refractory demyelinating episodes, including tumefactive presentations, particularly when initiated early [22].

When PLEX is contraindicated or unavailable, intravenous immunoglobulin (IVIG) has been reported as an alternative in selected cases, although evidence supporting its efficacy remains limited and inconsistent.

In rare instances of fulminant lesions causing significant intracranial pressure or signs of impending herniation—features that may mimic high-grade neoplasms—more aggressive intervention may be required. This can include cytotoxic immunosuppression with agents such as cyclophosphamide and, in exceptional cases, neurosurgical decompression. Such measures are generally reserved for severe, treatment-refractory variants of demyelinating disease [22].

#### 3.15.2. Subacute Phase: Risk Stratification and Transition

Following acute stabilization, the clinician must assess the likelihood of future disease activity. Patients who meet diagnostic criteria for multiple sclerosis, either through evidence of dissemination in space and time or by fulfilling CSF and imaging criteria, should be considered for initiation of an appropriate disease-modifying therapy (DMT). Given that tumefactive presentations may be aggressive, some experts advocate early use of high-efficacy agents, such as anti-CD20 monoclonal antibodies or natalizumab. The decision to initiate therapy should be individualized, considering patient age, comorbidities, overall lesion burden, and patient preferences [21].

Conversely, in patients without a prior history of demyelinating events, with negative CSF oligoclonal bands and no new lesions on follow-up imaging, a monophasic course remains possible. In such cases, a period of close observation may be reasonable, particularly in older individuals. This approach requires structured follow-up with serial MRI, typically every 3 to 6 months during the first year, followed by annual imaging if clinical and radiologic stability is maintained [23].

If serologic testing demonstrates AQP4-IgG or MOG-IgG positivity, the therapeutic strategy should be reconsidered. These antibodies are associated with neuromyelitis optica spectrum disorder (NMOSD) and MOG-associated disease, both of which have distinct immunopathologic mechanisms compared to MS [24]. Importantly, standard MS therapies may be ineffective or even detrimental in these conditions. In such scenarios, long-term management is typically based on immunosuppressive regimens aimed at relapse prevention [25].

#### 3.15.3. Maintenance Phase: Disease Control and Therapeutic Tailoring

In patients with confirmed MS, long-term management with disease-modifying therapies (DMTs) should follow established treatment protocols, with surveillance MRI performed every 6 to 12 months and regular clinical assessment for relapse or disability progression. The occurrence of TMS should not automatically be interpreted as an indicator of a more aggressive long-term course. However, in selected cases, early initiation of high-efficacy agents may be considered to reduce the initial inflammatory burden. In patients who achieve sustained remission without radiologic activity, careful de-escalation under close specialist supervision may be appropriate [26,27].

Two important therapeutic pitfalls deserve particular attention. First, rebound demyelination has been described following discontinuation of certain DMTs. Fingolimod withdrawal, in particular, has been associated with new or recurrent tumefactive demyelinating lesions; therefore, abrupt cessation should be avoided, and a structured transition strategy between therapies is recommended. A similar phenomenon has been reported after natalizumab discontinuation [26,27]. In both settings, prompt recognition and timely escalation of immunomodulatory therapy are essential.

Supportive care remains important throughout all phases of management. Seizures, which are more commonly associated with large cortical or subcortical lesions, should be treated with appropriate antiepileptic therapy. Persistent cognitive or motor deficits may warrant formal rehabilitation. Residual edema may be managed with gradual steroid tapering or, in selected cases, adjunctive agents such as acetazolamide. Overall, the management of TMS requires a dynamic, stage-specific approach tailored to the individual patient [24,26,27].

### 3.16. Prognosis

Long-term prognosis largely depends on whether the patient develops a relapsing demyelinating course. Most patients who experience relapse eventually develop lesions characteristic of classical MS, whereas a smaller proportion present with recurrent tumefactive episodes. Importantly, patients who transition to MS after an initial TDL appear to have long-term disability outcomes comparable to those with typical MS [9].

#### 3.16.1. Monophasic Course

A substantial subset of patients experience a single tumefactive episode without subsequent clinical or radiologic activity. Reported rates of monophasic TDL vary considerably across studies and depend on cohort definitions and duration of follow-up [28]. Overall, a monophasic course has been described in approximately 35% to 60% of cases. In these patients, lesions often regress substantially or resolve completely, and most individuals achieve near-complete neurological recovery [10].

Follow-up MRI typically reveals either complete radiographic resolution or a small residual gliotic plaque [29]. Functionally, some patients may retain subtle cognitive or sensory deficits, although many regain independence. In one recent series, the median Expanded Disability Status Scale (EDSS) score improved from 4.3 at presentation to 3.0 at final follow-up, and 77% of patients remained relapse-free over a mean follow-up period of four years [29,30].

Patients who are MOG-IgG-positive and present with tumefactive lesions more commonly follow a monophasic course resembling acute disseminated encephalomyelitis (ADEM), particularly in pediatric and young adult populations [31]. These cases are often characterized by radiologic reversibility and favorable long-term outcomes.

In contrast, among seronegative TDLs, the absence of new lesions or recurrent clinical events over time may support classification as isolated demyelination rather than evolving MS [32,33].

#### 3.16.2. Relapsing Course and Conversion to MS

Conversely, a significant proportion of patients with TDLs remain at risk for subsequent demyelinating attacks. Reported relapse rates range from 45% to 46.6% [34,35]. These recurrences may present either as additional tumefactive lesions or as more typical demyelinating plaques.

The presence of periventricular or callosal lesions on initial MRI, as well as cerebrospinal fluid oligoclonal bands, increases the likelihood of progression to relapsing-remitting MS. In a large retrospective cohort, most patients who experienced relapse met formal diagnostic criteria for MS within one to two years of the initial event [17].

A smaller subset of patients demonstrates a distinct pattern of relapsing tumefactive demyelination without fully meeting criteria for MS or neuromyelitis optica spectrum disorder (NMOSD). These individuals may develop multiple large demyelinating lesions that are spatially separated and associated with stepwise accumulation of disability. While some eventually fulfil criteria for MS or are reclassified under recognized inflammatory syndromes, others remain diagnostically ambiguous and may represent an intermediate or overlapping disease phenotype [36].

#### 3.16.3. Prognosis in MOGAD and NMOSD

In patients whose tumefactive lesions fall within antibody-mediated demyelinating disorders, long-term outcomes differ substantially from classical MS. In AQP4-IgG-positive NMOSD, the presence of a solitary brain lesion does not confer a more favorable prognosis. Instead, NMOSD typically follows a relapsing course, with optic neuritis and transverse myelitis serving as the principal drivers of long-term disability [28]. Lifelong immunosuppressive therapy is generally required to reduce relapse risk.

In contrast, tumefactive lesions associated with MOG-associated disease (MOGAD), although sometimes severe at presentation, are more frequently monophasic or only infrequently relapsing. With appropriate treatment, typically acute immunosuppression followed by individualized maintenance strategies, many patients maintain functional independence and experience low levels of permanent disability [37]. When relapses occur, they more commonly involve the optic nerves or spinal cord rather than recurrence of large cerebral tumefactive lesions.

### 3.17. Long-Term Outcomes and Considerations

Several factors influence long-term prognosis. Lesion burden on follow-up MRI serves as a useful surrogate marker of ongoing disease activity. Approximately 90% of patients with monophasic TDLs demonstrate radiologic quiescence over time, with no new lesions detected beyond the initial event. In contrast, patients with a relapsing course tend to accumulate new lesions on serial imaging.

Involvement of eloquent cortical regions has important functional implications. Although large lesions in these areas do not always result in persistent motor deficits, impairments in executive function and processing speed may remain evident at one-year follow-up [38]. Cognitive outcomes are particularly relevant, as large solitary lesions—especially those affecting eloquent cortex—can lead to lasting executive dysfunction, even in the absence of overt motor impairment, which may not be adequately captured by EDSS scores [39].

Although progression to a secondary progressive phase appears to be uncommon, it cannot be entirely excluded in cases of tumefactive-onset MS. Available data suggest that most patients who eventually develop MS remain within a relapsing-remitting phenotype for several years before any progression is observed [10]. Importantly, the initial severity or size of a tumefactive lesion does not necessarily predict a more aggressive long-term course [40].

Mortality associated with TDLs is rare in contemporary clinical practice. Fatal outcomes are typically limited to severe, fulminant presentations that fail to respond to medical therapy. Overall, the majority of patients, whether monophasic or relapsing, experience stabilization or partial recovery of neurological function over time, even in resource-limited settings [40].

### 3.18. Comparative Imaging of TDLs and Common Mimics

Large demyelinating lesions can radiologically mimic tumors or infections. Careful interpretation of MRI can suggest one diagnosis over another. Table 2 contrasts key imaging features of tumefactive demyelination versus high-grade glioma, primary CNS lymphoma, brain abscess, and acute disseminated encephalomyelitis (ADEM).

### 3.19. Tumefactive MOGAD and AQP4+ NMOSD: Distinguishing Features in the Differential Diagnosis

Two important alternative etiologies of tumefactive demyelinating lesions (TDLs) include myelin oligodendrocyte glycoprotein antibody-associated disease (MOGAD) and aquaporin-4 immunoglobulin G (AQP4-IgG)-positive neuromyelitis optica spectrum disorder (NMOSD). Both conditions may present with large, space-occupying brain lesions that radiographically resemble tumors. However, their underlying pathophysiology, imaging characteristics, and therapeutic approaches differ substantially from those of TMS and must be carefully distinguished to avoid misclassification [28].

#### 3.19.1. Clinical Presentation

Tumefactive MOGAD most commonly presents as a monophasic, acute disseminated encephalomyelitis (ADEM)-like syndrome, particularly in children and young adults. Episodes are often preceded by infection or, less commonly, vaccination. Patients may develop encephalopathy, headache, or seizures—features that are relatively uncommon in typical MS presentations [41]. The coexistence of optic neuritis or longitudinally extensive transverse myelitis (LETM) with a tumefactive brain lesion should raise suspicion for an antibody-mediated demyelinating syndrome, particularly ADEM or MOGAD.

In contrast, AQP4-IgG-positive NMOSD predominantly affects adult women and is classically characterized by recurrent episodes of optic neuritis and LETM. Although large cerebral lesions at disease onset are less common, brain involvement may emerge during subsequent relapses. When present, lesions frequently involve the diencephalon, brainstem, or hypothalamus. Cortical dysfunction is uncommon in NMOSD compared with MS. A hallmark clinical feature is area postrema syndrome, characterized by intractable nausea, vomiting, or hiccups. This presentation is highly specific for AQP4-related disease and is not typical of MS [42].

#### 3.19.2. MRI Characteristics

Neuroimaging plays a central role in differentiating these antibody-mediated disorders from TMS. In MOGAD, lesions often exhibit poorly defined or “fluffy” margins and frequently involve deep gray matter structures, as well as infratentorial regions, including the cerebellar peduncles and basal ganglia [43]. MOGAD-associated lesions may demonstrate substantial surrounding edema and can display an open-ring or cloud-like pattern of contrast enhancement. These imaging features, in the appropriate clinical context, can help distinguish MOGAD from classical MS [44].

In AQP4-IgG-positive NMOSD, brain lesions are commonly located adjacent to ventricular surfaces, particularly within the periependymal regions of the third and fourth ventricles. Lesions may also extend longitudinally within the brainstem or diencephalon. Contrast enhancement is often patchy or subtle, although in some cases the radiologic appearance may overlap with MS [43]. Key distinguishing features include characteristic lesion localization, relative sparing of the cortex, and the frequent presence of concomitant spinal cord or optic nerve involvement, which together facilitate differentiation from MS [44].

Two additional imaging markers may further aid distinction from MS. The central vein sign is frequently observed in MS plaques but is generally absent in both MOGAD and NMOSD. Similarly, the presence of a T2 hypointense rim is more characteristic of chronic MS lesions than of antibody-mediated demyelinating disorders [45,46].

#### 3.19.3. Cerebrospinal Fluid and Serological Testing

Serologic testing plays a pivotal role in distinguishing antibody-mediated demyelinating diseases from MS. MOGAD is defined by the presence of serum MOG-IgG, and NMOSD by AQP4-IgG detected using a cell-based assay; both antibodies are disease-specific and pathogenic. In contrast, MS remains a diagnosis of exclusion, typically supported by CSF-restricted oligoclonal bands (OCBs) and an elevated IgG index. OCBs are present in more than 90% of patients with MS but are uncommon in MOGAD and NMOSD [42].

In pediatric MOGAD, transient OCB positivity has occasionally been reported, although this finding remains rare [47].

Given the significant differences in disease course and treatment strategies, the presence of atypical imaging or CSF findings should prompt careful evaluation for MOG-IgG and AQP4-IgG antibodies, particularly in patients presenting with tumefactive lesions that resemble MS [48]. A positive antibody result allows reclassification of the underlying disorder and carries important therapeutic implications, as management strategies differ substantially from those used in MS [49].

#### 3.19.4. Prognosis and Treatment Implications

The natural history of MOGAD and NMOSD differs substantially from that of MS. Tumefactive MOGAD frequently follows a monophasic course, particularly in pediatric populations, and is associated with high rates of radiologic resolution. In one series, complete resolution of tumor-like MOGAD lesions was observed in up to 72% of cases [50]. Even when relapses occur, they tend to be less severe on average and generally respond well to immunotherapy.

In contrast, AQP4-IgG-positive NMOSD is a relapsing disorder associated with a significant risk of cumulative neurological disability if left untreated [51]. Standard MS therapies, including interferon-β and fingolimod, are not only ineffective in NMOSD but may worsen disease activity [52]. Early initiation of targeted immunosuppressive therapy, such as rituximab, inebilizumab, or tocilizumabis, is essential to reduce relapse risk and preserve neurological function.

Failure to distinguish these antibody-mediated demyelinating syndromes from MS may lead to inappropriate management. Misclassification as monophasic MS could result in undertreatment of NMOSD, whereas treating a monophasic MOGAD episode with long-term MS disease-modifying therapy may expose patients to unnecessary chronic treatment [48]. Therefore, accurate differentiation is not merely a diagnostic consideration but also a therapeutic imperative. Establishing the correct diagnosis enables timely initiation of appropriate immunotherapy while avoiding treatments that may be ineffective or potentially harmful [53].

### 3.20. Common Pitfalls

#### 3.20.1. Misdiagnosis Leading to Inappropriate Treatment

One of the most serious diagnostic errors is misclassifying a demyelinating lesion as a malignant neoplasm or abscess, or vice versa. A large, contrast-enhancing lesion that is inadequately evaluated may be prematurely attributed to multiple sclerosis, potentially delaying the diagnosis of glioma or lymphoma [54].

Conversely, primary central nervous system lymphomas can initially present with demyelination-like features and may show transient radiologic improvement following corticosteroid therapy. This temporary response can create a false impression of remission and delay definitive biopsy and diagnosis. Therefore, any discrepancy between clinical presentation and imaging findings should prompt careful reassessment before concluding that a lesion is demyelinating in origin [55].

#### 3.20.2. Incomplete Diagnostic Workup

Failure to perform essential ancillary investigations—such as cerebrospinal fluid (CSF) analysis and serum testing for aquaporin-4 and MOG antibodies—may compromise diagnostic accuracy. A negative antibody panel or absence of CSF oligoclonal bands should prompt expansion of the differential diagnosis rather than premature diagnostic closure [47].

Conversely, the presence of CSF-restricted oligoclonal bands provides strong support for MS [42]. Inadequate evaluation increases the risk of anchoring bias, potentially leading to misdiagnosis of MS when the underlying condition is an antibody-mediated demyelinating disorder such as NMOSD or MOGAD.

#### 3.20.3. Delayed Biopsy in Atypical Cases

Although an initial trial of corticosteroids may be reasonable in clinically stable patients, prolonged empiric therapy without diagnostic confirmation can be hazardous, particularly if the underlying pathology is infectious or neoplastic [16].

For example, treating a cerebral abscess as a tumefactive demyelinating lesion and initiating immunosuppression may allow the infection to progress. Similarly, failure to recognize an underlying neoplasm can delay appropriate oncologic management. If a lesion fails to regress, enlarges despite therapy, or demonstrates atypical imaging characteristics, early biopsy should be strongly considered.

#### 3.20.4. Over-Reliance on Corticosteroid Responsiveness

Although a favorable response to corticosteroids is a common feature of tumefactive demyelinating lesions, it is not pathognomonic [56]. Certain neoplasms, particularly primary CNS lymphomas, may demonstrate transient radiologic improvement following steroid administration. Similarly, lesions associated with substantial perilesional edema, regardless of the underlying etiology, may appear to regress after corticosteroid therapy.

In infectious processes, partial suppression of inflammatory changes may temporarily obscure the true diagnosis and delay appropriate investigation [57]. For this reason, steroid responsiveness should be interpreted within the broader clinical and radiologic context rather than considered diagnostic in isolation.

#### 3.20.5. Therapy Misalignment and Rebound Syndromes

Inappropriate initiation, continuation, or abrupt discontinuation of disease-modifying therapies (DMTs) may also lead to complications. Fingolimod withdrawal has been associated with rebound inflammatory activity, including the development of new tumefactive lesions. This rebound phenomenon may be misinterpreted as aggressive MS or progressive multifocal leukoencephalopathy (PML), particularly in patients previously treated with natalizumab [58].

To mitigate this risk, a carefully planned and monitored transition strategy between therapies is essential. Additionally, certain MS-specific treatments, such as interferon-β, may exacerbate antibody-mediated conditions like NMOSD, potentially leading to increased disease activity [59].

#### 3.20.6. Insufficient Long-Term Monitoring

Although some tumefactive presentations are monophasic, relapses have been documented even years after the initial episode. Premature discontinuation of follow-up after a single event increases the risk of delayed recognition of relapse or conversion to MS. Accordingly, structured clinical and radiologic monitoring is recommended for at least five years following the initial presentation.

Conversely, prolonged continuation of immunotherapy in patients who remain clinically stable and show no new radiologic activity should also be reconsidered. Periodic reassessment of the ongoing need for therapy is warranted to balance efficacy and long-term safety [34].

#### 3.20.7. Neglect of Alternative Diagnoses

In complex or atypical cases, alternative diagnoses must be carefully considered. In immunosuppressed patients, progressive multifocal leukoencephalopathy (PML) may present with large, non-enhancing lesions that can be radiographically mistaken for TDLs [60]. Other important considerations include tumefactive neurosarcoidosis, granulomatous infections, and paraneoplastic encephalitis.

A narrowly focused diagnostic approach may lead to inappropriate immunosuppression and potentially serious consequences. Maintaining a broad differential diagnosis is therefore essential, particularly in patients with atypical clinical or radiologic features [61,62].

#### 3.20.8. Diagnostic Rationale for Deferring Tissue Biopsy

In the present case, high-grade glioma and brain abscess were carefully considered in the differential diagnosis because of the lesion size, enhancement pattern, edema, and mass effect. However, these entities were considered less likely after multidisciplinary review of the clinical, radiologic, and cerebrospinal fluid findings. The open-ring enhancement pattern, with the incomplete portion directed toward the cortical surface, favored tumefactive demyelination over a neoplastic process. In addition, the absence of prominent central diffusion restriction made a pyogenic abscess less likely, as brain abscesses more typically demonstrate a smooth complete ring with marked central diffusion restriction due to purulent material. Supportive cerebrospinal fluid findings, including elevated IgG index, increased CNS IgG synthesis, and an atypical CSF-restricted oligoclonal band, further supported an inflammatory demyelinating process rather than infection or primary glial neoplasm.

Therefore, tissue biopsy was deferred because the available findings supported a demyelinating etiology, and the patient did not demonstrate clinical or radiologic features that mandated urgent tissue diagnosis. Nevertheless, glioma and brain abscess cannot be excluded with absolute certainty in the absence of histopathologic confirmation. Brain biopsy remains essential when imaging features are atypical, when there is progressive clinical deterioration, when the lesion fails to regress after appropriate corticosteroid therapy, or when infection or malignancy cannot be reasonably excluded.

#### 3.20.9. Role of Advanced MRI, Radiogenomics, and IDH Status

Advanced MRI techniques can be useful when conventional MRI findings are equivocal. Diffusion-weighted imaging, perfusion imaging, magnetic resonance spectroscopy, and PET may help distinguish high-grade glioma, lymphoma, abscess, and tumefactive demyelination by assessing lesion cellularity, vascularity, metabolic activity, and diffusion characteristics. In suspected glioma, IDH status has major diagnostic and prognostic implications under the contemporary WHO classification; however, IDH mutation status is a molecular marker and cannot be established by conventional MRI alone. Although MRI-based radiogenomic approaches have been explored to estimate molecular profiles noninvasively, current evidence remains inconclusive, and validated imaging criteria for reliable molecular prediction are not yet available. Therefore, advanced imaging may increase or decrease suspicion for high-grade glioma, but it does not replace histopathologic and molecular confirmation when tissue diagnosis is clinically indicated [63].

In the present case, the available conventional MRI features and cerebrospinal fluid profile favored tumefactive demyelination. Nevertheless, advanced imaging modalities such as perfusion MRI, MR spectroscopy, amino acid PET, or radiogenomic modeling were not available for direct comparison. Their absence is therefore acknowledged as a limitation, particularly because such modalities could have provided additional information regarding tumor vascularity, metabolic behavior, and molecular suspicion. This limitation further supports the need for close clinical and radiologic follow-up in cases managed without tissue confirmation.

#### 3.20.10. Limitations

This report has several limitations. First, as a single case report, its findings cannot be generalized to all patients with tumefactive demyelinating lesions. Second, the diagnosis was supported by clinical presentation, MRI characteristics, cerebrospinal fluid findings, and multidisciplinary interpretation; however, histopathologic confirmation was not obtained. Although brain biopsy is not required in every clinically stable patient with imaging features suggestive of demyelination, the absence of tissue diagnosis limits absolute diagnostic certainty. Third, not all advanced diagnostic modalities, such as magnetic resonance spectroscopy, perfusion imaging, or FDG-PET, were available for direct comparison in this case. Finally, the literature review was narrative rather than systematic and therefore may be subject to selection bias. Despite these limitations, the case emphasizes the importance of integrating radiologic, laboratory, and clinical data to avoid misdiagnosis and unnecessary invasive interventions in suspected tumefactive multiple sclerosis.

## 4. Conclusions

Tumefactive multiple sclerosis represents a challenging demyelinating phenotype that often poses significant diagnostic uncertainty. Its clinical and radiologic overlap with neoplastic and infectious conditions underscores the importance of a comprehensive, multimodal diagnostic strategy that incorporates advanced MRI techniques, cerebrospinal fluid analysis, and serologic testing for MOG-IgG and AQP4-IgG antibodies.

Although emerging biomarkers continue to refine diagnostic precision, clinical response to corticosteroid therapy remains an important, but not definitive, component of the diagnostic process. Brain biopsy retains a role in atypical or treatment-refractory cases where diagnostic ambiguity persists.

Despite the often alarming initial presentation of tumefactive lesions, prognosis is generally favorable when timely recognition and appropriate immunotherapy are achieved. Many patients experience substantial neurological recovery, and a significant proportion follow a monophasic course.

Ongoing advances in neuroimmunology and biomarker development are expected to further clarify disease classification, optimize therapeutic decision-making, and reduce both overtreatment and unnecessary surgical intervention. Continued research will be essential to improve diagnostic accuracy and refine management strategies for this complex and evolving disorder.

## Figures and Tables

**Figure 1:**
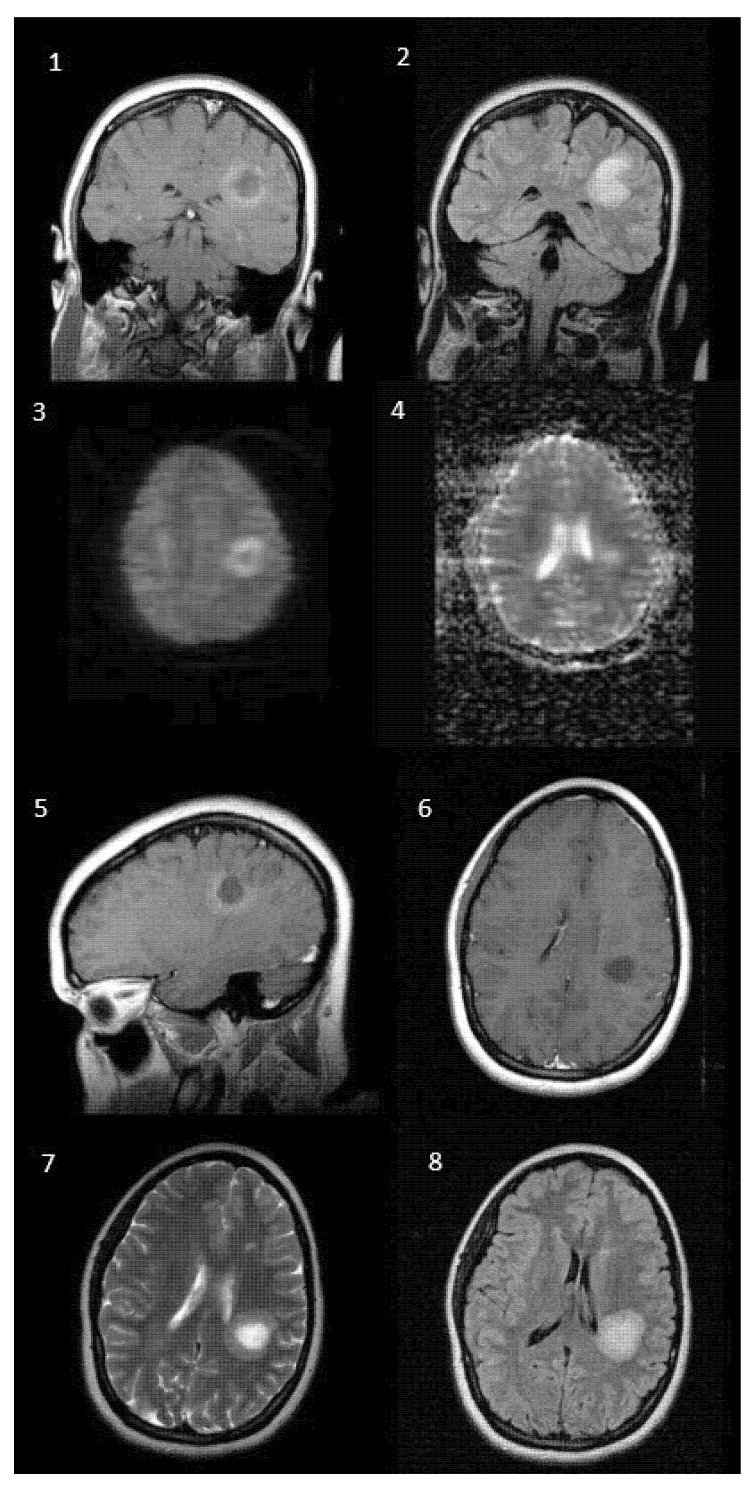
Brain MRI. Image 1. Coronal T1-weighted post-contrast MRI showing a left fronto-parietal intra-axial open-ring enhancing lesion, also referred to as the horseshoe sign, surrounding a non-enhancing hypointense central region. Image 2. Coronal FLAIR MRI demonstrating a left fronto-parietal hyperintense lesion with extensive surrounding FLAIR hyperintensity, consistent with vasogenic edema. Images 3 and 4. Axial diffusion-weighted imaging (DWI) and apparent diffusion coefficient (ADC) map demonstrating peripheral signal abnormality without marked central diffusion restriction. Images 5 and 6. Sagittal and axial T1-weighted post-contrast MRI demonstrating a well-defined intra-axial left fronto-parietal rounded lesion with peripheral open-ring enhancement and a relatively hypointense central core. Image 7. Axial T2-weighted MRI showing a well-defined hyperintense central area with a thin hyperintense peripheral margin. Extensive T2 hyperintense perilesional signal is present within the left fronto-parietal deep white matter, compatible with edema. Mild mass effect is present, with partial compression of the left lateral ventricle. Image 8. Axial FLAIR MRI showing a hyperintense fronto-parietal lesion with a slightly hypointense rim corresponding to the low-signal component seen on T1. Surrounding FLAIR hyperintensity involves the left frontal white matter, consistent with edema.

**Table 1:** Laboratory results.

Component	Result 2 May 2010	Reference Range
Color, CSF	Colorless	
Clarity, CSF	Clear	
RBC	0	
WBC	1	
Neutrophil %	1%	
Lymphocytes %	78%	
Monocytes %	21%	
Glucose	75	
Protein	34	
Immunoglobulin G CSF	3.2	0.6–4.2 mg/dL
Albumin CSF	16.1	9.3–31.3 mg/dL
Serum IgG	972	717–1411 mg/dL
Serum Albumin	3510	3276–4819 mg/dL
IgG/Albumin Ratio	0.20 (H)	0.06–0.17
CNS IgG Synthesis	4.9 (H)	0–3.0 mg/day
IgG Index	0.72 (H)	0–0.61
HIV	Negative	

**Table 2:** Condensed imaging discriminators: TDL vs. common mimics.

Feature	Tumefactive MS (TDL)	High-Grade Glioma (GBM)	Primary CNS Lymphoma	Brain Abscess	ADEM (Polyfocal)
Enhancement	Open/incomplete ring	Heterogeneous ± thick ring	Solid, homogeneous (±ring if necrosis)	Thin, smooth complete ring	Patchy/”fluffy”, often faint
Perfusion (rCBV)	Low/normal	High (neoangiogenesis)	Normal–mild ↑ (↓ than GBM)	Low (necrotic core)	Low/normal
DWI/ADC	No core restriction; rim restriction possible	Mixed; no uniform restriction	Restricted (low ADC)	Central restriction (pus)	No significant restriction
MRS	↑ Cho, ↓ NAA; lipid/lactate possible; Cho/NAA < tumors	Marked ↑ Cho, ↓ NAA; ±lactate/lipid	↑ Cho, ↓ NAA; prominent lipids	Amino-acid peaks (acetate/succinate); high lactate	Mild ↑ Cho, ↓ NAA; reversible pattern
Mass effect/Edema	Mild for size	Marked; extends beyond margins	Variable–moderate	Disproportionately extensive	Moderate–severe, often diffuse
Steroid response	Dramatic, rapid	Edema ↓, lesion persists	Transient shrinkage (pitfall)	Minimal without antibiotics	Good; often full resolution

↑: elevated; ↓: reduced.

## Data Availability

The data presented in this study are available on request from the corresponding author due to privacy concerns.
